# Acquired hemophilia associated with vedolizumab in a patient with hemorrhagic rectocolitis

**DOI:** 10.1097/BS9.0000000000000138

**Published:** 2022-10-26

**Authors:** Thomas Schiestel

**Affiliations:** Regional Pharmacovigilance Center, Fernand Widal Hospital, APHP, Paris, France

## To The Editor:

Hemorrhagic rectocolitis is an autoimmune disease that affects the gastrointestinal tract. Treatment of the disease includes 5-aminosalicylic acid drugs such as mesalazine at first-line therapy, with a possible association of corticosteroids. In the case of severe inflammation, treatment with purine analogue (azathioprine), TNF-alpha inhibitor (infliximab, adalimumab), or integrin inhibitor (vedolizumab) may be initiated.^1^

Vedolizumab is a monoclonal antibody that selectively targets alpha4-beta7 integrin used for Crohn disease and hemorrhagic rectocolitis.^2^ Whereas the efficacy and the security of vedolizumab seem to be well described since the commercialization of the drug,^2,3^ we notice its hematologic toxicity is poorly documented. We herein describe the first case of acquired hemophilia in a patient with hemorrhagic rectocolitis while on vedolizumab.

The patient is a 51-year-old female diagnosed with hemorrhagic rectocolitis in April 2014 and with no other notable medical history. More specifically, she has no familial history of hemophilia nor Van Willebrand disease. Treatment with mesalazine was initially started in 2015 before being withdrawn because of the inflammatory condition of the patient refractory to salicylates drugs. The patient began a treatment with vedolizumab in February 2019 after a medication attempt with adalimumab between 2018 and 2019. Three months after the start of vedolizumab, in May, the patient noticed the occurrence of some episodes of epistaxis, gingivorrhagia, and a rectal bleeding which is estimated by herself as more important than the ones she used to suffer from with her disease. Thereafter, spontaneous hematomas of the 2 hands, forearms, and arms are observed by the patient. She was admitted to the intensive care unit 6 days after the occurrence of the symptoms and it was concluded to an absence of hemodynamic instability, mucosal bleeding, central nervous system bleeding, hematuria, and hemorrhagic stools. Hemostatic tests showed an isolated elongation of the activated partial thromboplastin time to 2.6 times compared with the control (normal < 1.20), a normal prothrombin ratio at 90%, a normal plasma thrombin time, a hemoglobin at 13.3 g/dL and a normal platelet level at 306G/L. Then, a diagnosis of acquired hemophilia was made. Three days after, more examinations highlight the previous observations: a Factor VIII inhibitor titer positive at the high level of 15.6 BU (Bethesda Units), a low activity of factor VIII within only <1%, a von Willebrand factor risen to 142%, no anomaly regarding the complement system (C3/C4/CH50), a negative rheumatoid factor, a normal prothrombin time and finally a normal fibrinogen activity. Besides, there are no cardiovascular, respiratory, neurological, abdominal, or lymph node abnormalities. The clinical condition of the patient was favorable 5 days after treatment with rituximab, solumedrol, tranexamic acid, and eptacog alfa. At this time, only activated partial thromboplastin time value was still abnormal (1.83), and factor VIII activity was not being measured. It finally increased to 43% after 15 days, before the patient leaves the hospital.

Managing a case of acquired hemophilia may be an arduous challenge. Treatment generally consists of the lessening of the hemorrhagic syndrome and the inhibition of the autobodies activity. Bleeding may be controlled by recombinant factor VII or VIII and the elimination of autobodies may involve the use of immunosuppressive drugs such as corticosteroids, rituximab, or cyclophosphamide.^4,12^

The mechanism of acquired hemophilia is not completely identified but involves a neutralizing action of factor VIII by polyclonal or monoclonal antibodies.^4,5^ A small proportion of acquired hemophilias are drug-induced.^6,7^ Among the drugs best known for causing acquired hemophilia we can quote antibiotics from the penicillin family, antiplatelet agents, and non-steroid anti-inflammatory drugs.^7,8^ Nevertheless, the literature also mentions cases of hemophilia induced by monoclonal antibody: alemtuzumab (anti-CD52),^9^ adalimumab (TNF-alpha inhibitor),^10^ omalizumab (IgE inhibitor)^11^ or Nivolumab (PD-1 inhibitor).^12^ Anticytokine therapy is also concerned such as etanercept for rheumatoid arthritis.^13^ The autoimmune condition of the patients seems to play a role in the occurrence of acquired hemophilia, particularly in the case of systemic lupus erythematosus.^14,15^ However, we did not find in the literature any mechanism that could explain the link between acquired hemophilia and hemorrhagic rectocolitis. The other non-medicamentous etiologies widely mentioned are pregnancy, genetic predisposition, and malignancies.^6,15^

In this case, the association between the occurrence of acquired hemophilia and vedolizumab therapy was considered possible, especially because of a compatible time to onset and the recovery of the patient after discontinuation of treatment. Hemostatic tests emphasized the diagnosis of acquired hemophilia because of the increase of activated partial thromboplastin time and the collapse of factor VIII among others. No other etiology was found. However, we hardly can exclude a potential role of hemorrhagic rectocolitis in the occurrence of hemophilia. Also, we regret to not have any further information about the long-term evolution of hemophilia, whereas she was greatly recovering within a few days after the symptoms happen. We underline the originality of this case insofar, to our knowledge, there is no case described in the literature of acquired hemophilia induced by integrin alpha4–beta7 inhibitors such as vedolizumab but also natalizumab, which is prescribed for multiple sclerosis and Crohn disease.

New studies are expected in order to explore the pharmacological action behind drug-induced acquired hemophilia and, consequently, to upgrade the recommendations relating to the management of the potential life-threatening conditions in connection with this secondary autoimmune disease.

## ACKNOWLEDGMENTS

We thank the Medical Intensive Care Department of Hôpital Saint-Louis (1, avenue Claude Vellefaux – 75475, Paris Cedex 1, Paris, France) for notifying us of this original case.
